# Supplementary material for: A method for measuring mitochondrial DNA copy number in pediatric populations
Source: Front Pediatr. 2024 Jun 13;12:1401737. doi: 10.3389/fped.2024.1401737 (PMC11208623; doi:10.3389/fped.2024.1401737)
Supplement: Supplementary file 1 [file Datasheet1.pdf]

1 Supplementary Material

Inter and intra-day validation of the commercial NovaQUANT mtDNA qPCR assay

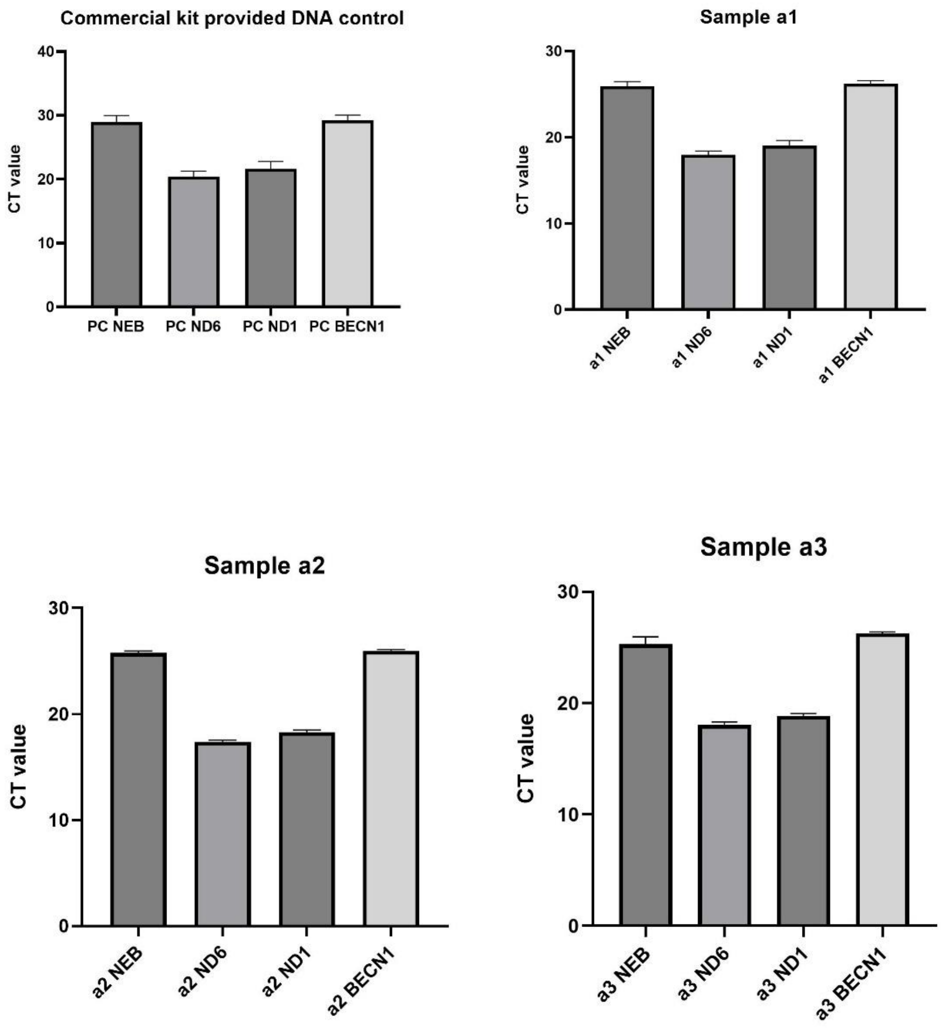

Figure s1. Mitochondrial DNA copy number assay validation. The qPCR assay utilizes two nuclear (NEB, BECN1) and two mitochodnrial genes (ND1, ND6). The graphs above show the average CT values ± SD from three separate validation runs. In each run the individual DNA sample was run in triplicate. Positive control (PC) is a DNA sample provided by the kit manufacturer.

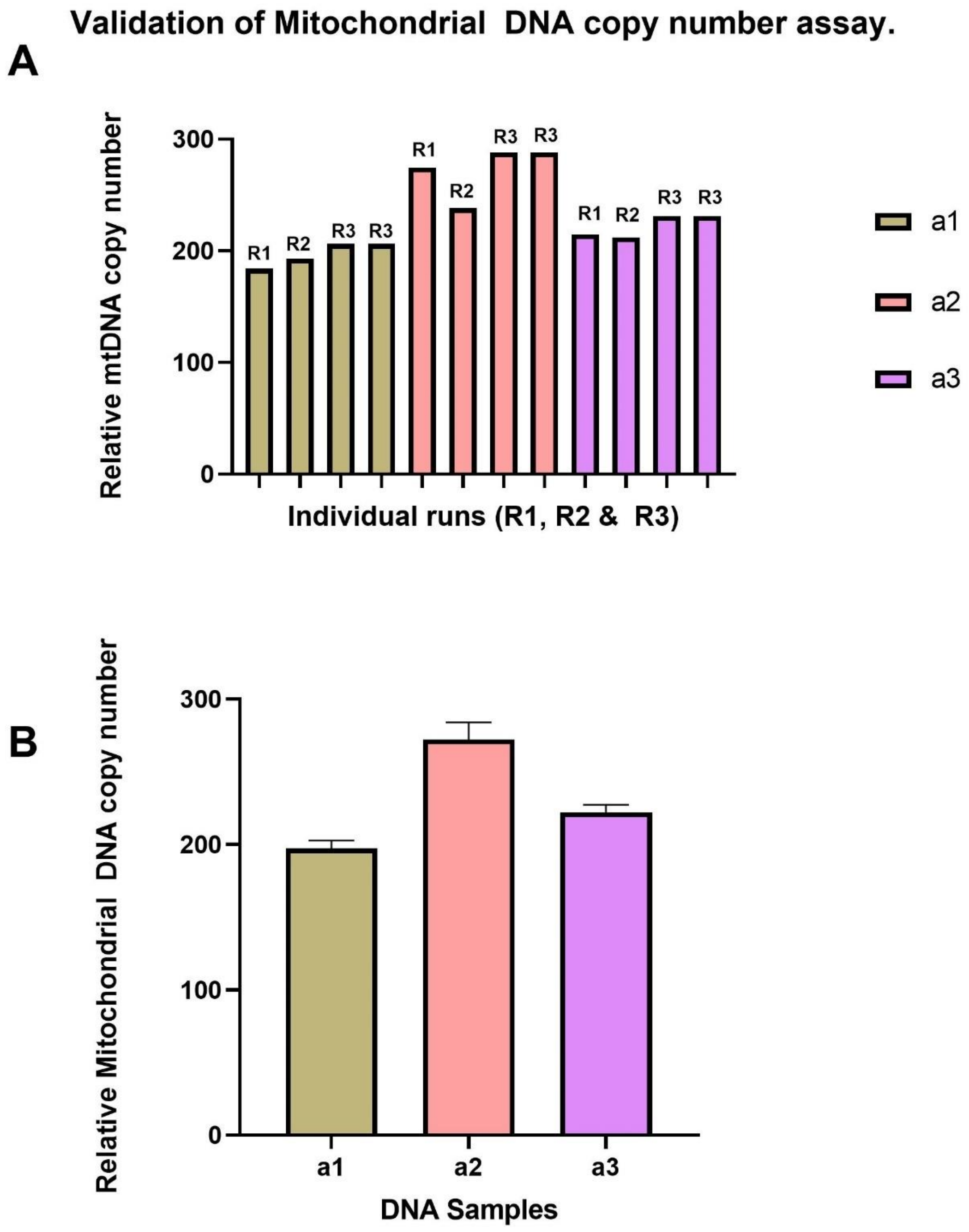

Figure s2. Panel A - DNA samples were assayed in 3 separate validation runs (R1, R2, R3). R1 and R2 are inter-day validation runs. R3 is the intra-day validation run. In the third validation run (R3,intra-day), each sample was run twice (in triplicate). Panel B - Average of all runs. The graphs above show the average mitochondrial DNA copy number ± SD.

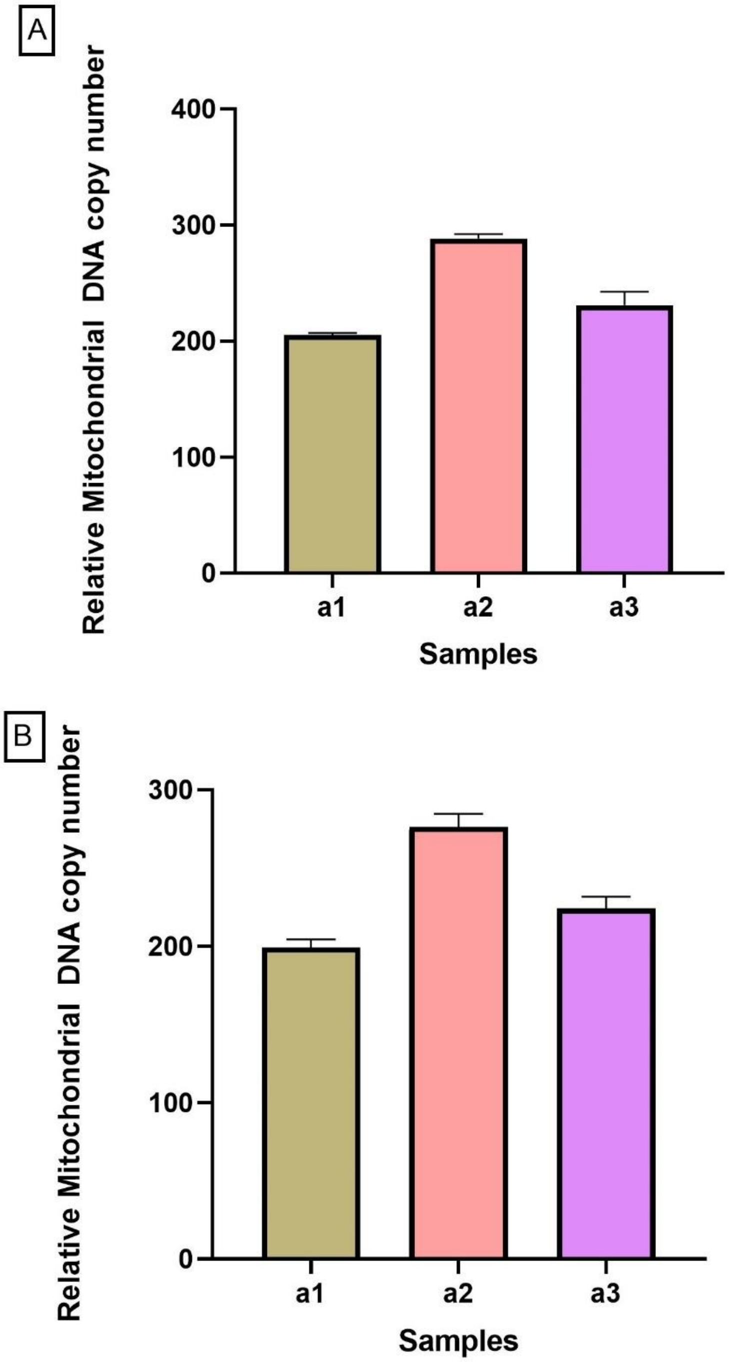

Figure s3. The graphs above show validation results from intra (A) and inter (B) runs. Inter-day results show relative mitochondrial DNA copy number ± SD from samples run consecutively on two separate days on different plates in triplicate. Intra-day results show relative mitochondrial DNA copy number ± SD from samples run TWICE on the same plate in triplicate.

Inter- and intra-day validation of our qPCR mtDNAcn assay

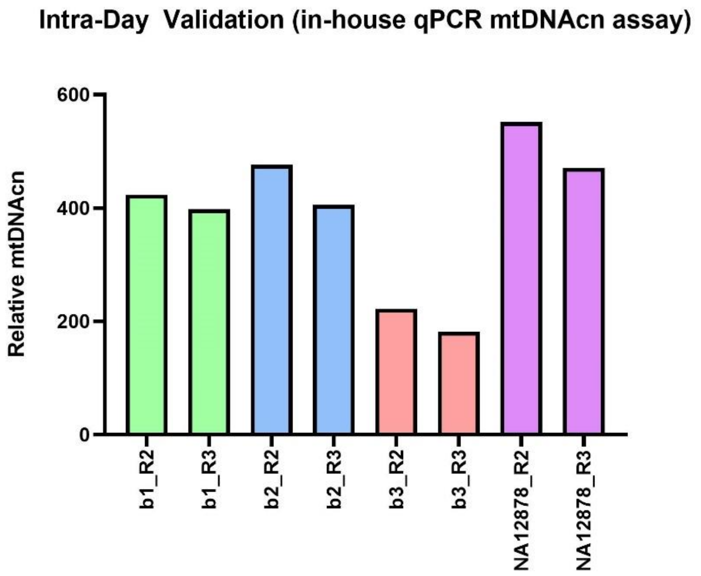

Figure s4 shows results of INTRA-day validation of in-house qPCR mtDNAcn assay. Run2 (R2) was carried out in the morning, and RUN3 (R3) was carried out in the afternoon of the same day. The different colored bars represent samples b1, b2, b3 and internal standard DNA sample - NA12878 respectively.

Inter-Day validation (In-house qPCR mtDNAcn assay).

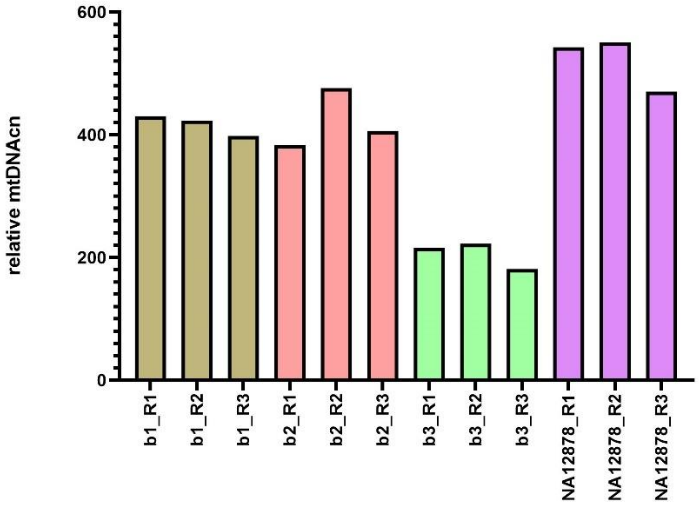

Figure s5 shows INTER-DAY validation of in-house qPCRmtDNA assay. Run1 (R1) and Run2 (R2) were carried out on consecutive days. Run 2 and Run3 (R3) were carried out on the same day. The different colored bars represent samples b1, b2, b3 and internal standard DNA sample - NA12878 respectively.

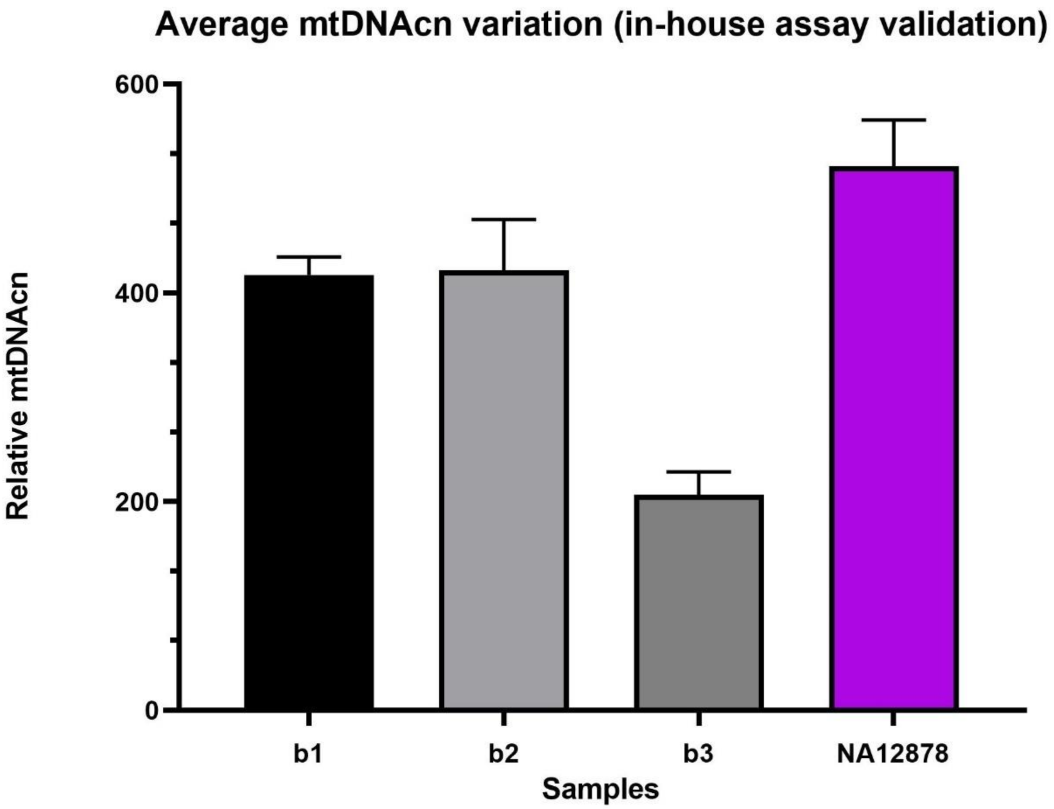

Figure s6 shows the average mtDNAcn and standard deviations from three separate runs.

RNA quality and quantity of extracted RNA from ACEs study

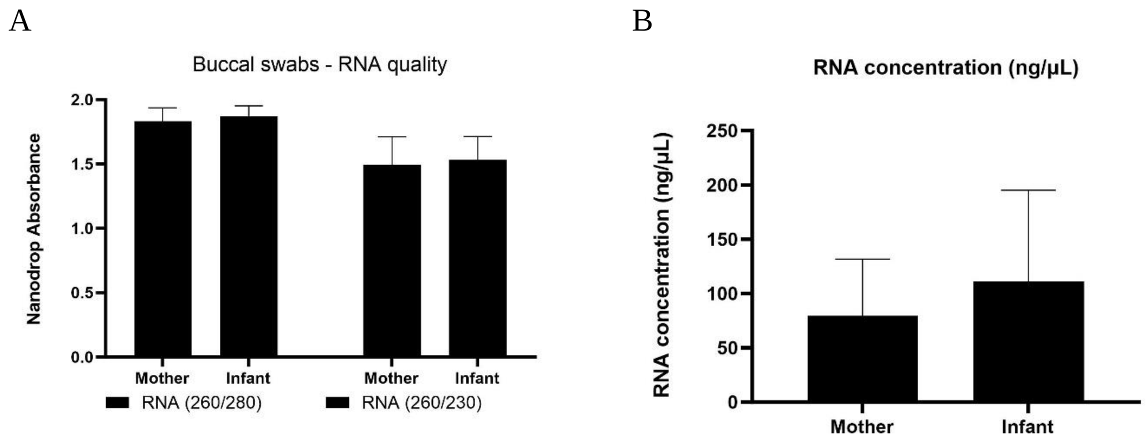

Figure s7 *RNA quality (panels A) and quantity (Panel B) of 164 mother and 164 infant samples from the ACEs cohort.*
